# Effects of stroboscopic vision training on visuomotor reaction and soccer-specific reactive agility in male collegiate soccer players: a randomized controlled intervention study

**DOI:** 10.3389/fphys.2026.1897512

**Published:** 2026-07-20

**Authors:** Congcong Weng, Dongxu Gao, Xiushen Dong, Saijun Xu, Jieru Jiang, Jiayu Wang

**Affiliations:** 1 Department of Physical Education and Military Training, Jiaxing University, Jiaxing, China; 2 Division of Sports Science and Physical Education, Tsinghua University, Beijing, China; 3 School of Physical Education, Hunan Normal University, Changsha, China; 4 Department of Physical Education, Jiaxing Nanhu University, Jiaxing, China; 5 School of Physical Education, Minnan Normal University, Zhangzhou, China; 6 School of Physical Education, Hubei University, Wuhan, China

**Keywords:** perception–action coupling, reactive agility, soccer, stroboscopic vision training, visuomotor reaction performance

## Abstract

**Background:**

Soccer is an open-skill sport in which players must rapidly process visual cues and transform them into appropriate motor actions under changing contextual constraints. Stroboscopic vision training has been proposed as a method for enhancing visuomotor processing and sport-related performance, but its value as an embedded visual constraint within regular soccer-specific training remains unclear.

**Methods:**

This single-site, two-arm, parallel-group randomized controlled intervention study examined whether a 6-week stroboscopic vision training program combined with soccer-specific practice could improve visuomotor reaction performance and soccer-specific reactive agility in male collegiate soccer players. Thirty-six players were randomly assigned to either a stroboscopic training group or a non-stroboscopic group, with 18 participants in each group. Both groups completed the same soccer-specific training tasks three times per week. The stroboscopic training group wore Senaptec Strobe eyewear with the stroboscopic mode activated, whereas the non-stroboscopic group wore visually similar clear non-stroboscopic eyewear that did not produce visual occlusion or stroboscopic restriction. Outcome assessors were blinded to group allocation during pre- and post-intervention testing. Simple motor time, simple lower-limb visuomotor reaction time, complex reaction speed, with-ball reactive agility time, and without-ball reactive agility time were assessed before and after the intervention. Intervention effects were primarily evaluated using linear mixed-effects models with group, time, and group × time interaction as fixed effects and participant ID as a random intercept. Training experience and weekly training time were included as covariates. ANCOVA was also conducted as an adjusted analysis, with post-test values as dependent variables and corresponding pre-test values and training-related variables as covariates.

**Results:**

The linear mixed-effects models showed significant group × time interactions for all five outcomes, indicating greater pre-to-post improvements in the stroboscopic training group than in the non-stroboscopic group. Compared with the non-stroboscopic group, the stroboscopic training group showed larger reductions in simple motor time, simple lower-limb visuomotor reaction time, complex reaction speed, with-ball reactive agility time, and without-ball reactive agility time. The ANCOVA results were consistent with the primary analysis, showing significantly lower adjusted post-intervention values in the stroboscopic training group across all outcomes after controlling for baseline performance, training experience, and weekly training time. These findings suggest that adding stroboscopic vision training to soccer-specific practice may provide additional short-term benefits in visuomotor reaction efficiency and soccer-specific reactive agility.

**Conclusions:**

Compared with the same soccer-specific training performed with visually similar clear non-stroboscopic eyewear, adding stroboscopic vision training may further improve visuomotor reaction efficiency and soccer-specific reactive agility in male collegiate soccer players. This approach may be used as an auxiliary strategy for developing soccer-specific reactive ability, although further studies are required to determine whether these gains are retained over time and transferred to match play.

## 1 Introduction

Soccer performance is not determined by a single ability, but by the integrated contribution of multiple technical, tactical, physical, and psychological factors (Stølen et al., 2005). Within this multidimensional performance structure, perceptual-cognitive and visuomotor abilities are particularly important in open-skill and time-constrained match situations. As a dynamic open-skill sport, soccer requires players to rapidly pick up relevant information from the ball, teammates, opponents, and spatial configurations, and then make directional decisions and execute actions within very short time windows (Vaeyens et al., 2007; Roca et al., 2013). Among the multiple abilities that contribute to soccer performance, reactive agility is therefore an important capacity linking external-cue perception with movement execution. Reactive agility is not simply a matter of acceleration or pre-planned change-of-direction speed; it also reflects the ability to perceive information, select an appropriate response, and act according to external cues (Sheppard and Young, 2006; Pojskic et al., 2018).

Agility is important not only in team sports such as soccer, basketball, and handball, but also in individual open-skill sports such as badminton; however, compared with individual sports, soccer requires players to make response selections and movement adjustments within continuously changing interactions among teammates, opponents, and space (Phomsoupha and Laffaye, 2015). Therefore, in soccer, reactive agility particularly emphasizes the ability to rapidly transform visual information into response selection and soccer-specific movement adjustments under complex external-cue constraints. Evidence from previous research shows that reaction speed and reactive agility are strongly related to competitive level and soccer-specific performance, and these variables can help differentiate players with different performance capacities (Nimmerichter et al., 2016; Trajković et al., 2020; Andrašić et al., 2021). Moreover, athletes at higher levels usually demonstrate superior perceptual-cue utilization, response accuracy, and reaction time, suggesting that soccer-specific reactive ability depends on visual processing, response selection, and perception–action coupling (Savelsbergh et al., 2002; Farrow and Abernethy, 2003; Mann et al., 2007; Klatt and Smeeton, 2022). Accordingly, how to improve visuomotor reaction efficiency and soccer-specific reactive agility through training methods with sport-specific contextual constraints provides the problem basis for the present study.

Stroboscopic vision training has recently attracted attention as a potential method for enhancing perceptual-cognitive functions and sport-specific performance. In this form of training, athletes usually complete motor tasks while visual input is intermittently restricted, with the goal of improving subsequent performance when normal visual conditions are restored (Wilkins and Appelbaum, 2020). Such periodic visual restriction increases the requirement to identify key cues, process visual information for action, and regulate movement control. Consequently, SVT may improve the efficiency of visual information use and reinforce perception–action coupling (Appelbaum and Erickson, 2018; Wilkins and Appelbaum, 2020). Recent systematic reviews and meta-analyses have suggested that SVT is associated with reductions in reaction time and may benefit action-related outcomes such as reactive agility, general agility, and visuomotor performance (Dingirdan Gultekinler et al., 2025; Wang et al., 2025). Thus, SVT represents a practical training option for developing visuomotor reaction ability in open-skill sports.

Within open-skill and team ball sports, SVT research has increasingly shifted from general visual function tests toward sport-related response outcomes, including reactive agility, visuomotor reaction, coordination, and change-of-direction performance. Volleyball studies have reported improvements in visual function, visuomotor performance, and reactive agility in young athletes, as well as faster visuomotor responses during goal-directed actions after SVT (Zwierko et al., 2023, 2024a). Basketball research has likewise shown that combining SVT with sport-specific practice can improve coordination, change-of-direction ability, and decision-making performance (Li et al., 2026). These findings suggest that SVT may be useful in team ball sports that require rapid identification of external cues and immediate motor execution. Nevertheless, the magnitude and direction of its effects may depend on the sport, the structure of the training task, and the selected outcome measures (Luo et al., 2025). Therefore, although evidence from other team ball sports provides a rationale for applying SVT, it cannot substitute for soccer-specific evidence on visuomotor reaction and reactive-agility outcomes.

In contrast to the growing intervention evidence in volleyball and basketball, fewer studies have specifically examined whether SVT improves soccer-specific reactive agility (Beavan et al., 2021; Palmer et al., 2022; Zwierko et al., 2024b). Accordingly, although the available evidence supports the potential application of SVT, its practical value as an embedded visual constraint within regular soccer-specific training remains to be further clarified. Given the high demands of soccer competition for rapid visual judgment, response selection, and immediate action execution, further investigation of embedded SVT for soccer players remains warranted.

On the basis of this background and the identified research gap, the present study investigated the effects of stroboscopic vision training combined with soccer-specific practice on visuomotor reaction performance and soccer-specific reactive agility in male collegiate soccer players. We hypothesized that stroboscopic vision training would produce larger improvements in these outcomes than conventional soccer-specific training performed under visually similar clear non-stroboscopic eyewear.

## 2 Methods

The present study was a single-site, two-arm, parallel-group randomized controlled intervention study with a pre-test/post-test design. Participants were randomly allocated to either a stroboscopic training group or a non-stroboscopic group at a 1:1 ratio. Both groups completed the same 6-week soccer-specific training program, with the only planned between-group difference being whether stroboscopic visual occlusion was activated during training.

### 2.1 Participants

A convenience sample of 36 male collegiate soccer players was recruited. All participants held a valid Chinese National Level-1 Athlete certificate and had competed in the University Men’s Group A National Finals of the National Youth Campus Football League, a national-level competition within China’s highest-level campus soccer competition system. Goalkeepers were not included, and all participants were outfield players, comprising 16 defenders, 14 midfielders, and six forwards. Based on the team roster and official match records from the most recent competitive season before the intervention, 16 players were classified as starters and 20 as substitutes. Following eligibility screening, participants were randomly allocated in a 1:1 ratio to the stroboscopic training group or the non-stroboscopic group, with 18 players in each group. An *a priori* sample size calculation was performed using G*Power 3.1.9.7 to determine the minimum sample size required for a 2-group × 2-time-point intervention design. The analysis was based on the F test family, using the repeated-measures ANOVA model for within–between interaction effects. The number of groups was set to 2, the number of measurements was set to 2, the effect size was set to a medium level (f = 0.25), the significance level was set at α = 0.05, and the desired statistical power was set at 1 − β = 0.80. The correlation among repeated measures was set to 0.50, and the nonsphericity correction was set to ϵ = 1.00. The calculation indicated that a minimum total sample size of 34 participants was required. Considering the possibility of absence, injury, or incomplete data during the training period, 36 participants were finally recruited and allocated to the two groups at a 1:1 ratio (Faul et al., 2007).

Participants were eligible if they met all of the following criteria: (a) male collegiate soccer players; (b) possession of a valid Chinese National Level-1 Athlete certificate; (c) participation in the University Men’s Group A National Finals of the National Youth Campus Football League before study enrolment; (d) regular engagement in systematic soccer training; (e) normal or corrected-to-normal vision and the ability to correctly identify the visual stimuli presented during testing and training; (f) the physical capacity to safely complete the visuomotor and soccer-specific reactive-agility assessments; and (g) voluntary participation with written informed consent. These criteria were established with reference to previous studies involving high-level soccer players and stroboscopic vision training (Pojskic et al., 2018).

Participants were excluded if they: (a) were goalkeepers; (b) had a history of epilepsy, seizures, migraine, marked photosensitivity, or any other condition that made stroboscopic visual stimulation inappropriate; (c) had a severe visual impairment, neurological disorder, or vestibular dysfunction that could interfere with visual-stimulus identification or visuomotor performance; or (d) had a lower-limb injury that could affect sprinting, change-of-direction movements, or completion of the reactive-agility tests. These safety-related exclusion criteria were based on previous stroboscopic vision training studies and methodological recommendations (Appelbaum et al., 2012; Wilkins and Appelbaum, 2020; Zwierko et al., 2024a).

Before data collection, all participants received information about the study aims, testing procedures, possible risks, and data use, and they agreed voluntarily to participate. Written informed consent was obtained before the study began, including permission to use the testing data for research analysis. Throughout the study, personal information and test data were anonymized and used only for statistical analysis in this project. The protocol was approved by the Ethics Committee of the Medical College of Jiaxing University. The ethics approval number was TY-2026043. Trial registration: ISRCTN17651511.

### 2.2 Outcome measures and testing procedures

#### 2.2.1 Complex reaction speed

Complex reaction speed was assessed with the Determination Test (DT) from the Vienna Test System. This test is a multi-stimulus reaction task designed to evaluate reaction speed, sustained attention, and tolerance to reactive stress when visual and auditory stimuli change rapidly. Participants responded as quickly as possible with the appropriate hand or foot response according to different colored visual stimuli and different auditory tones (Teteris et al., 2025). The testing setup and stimulus–response arrangement for the complex reaction speed test are shown in **Supplementary Figure 1A**.

Testing was completed in a quiet indoor environment. Participants sat facing the screen and used the response panel and foot pedals supplied with the Vienna Test System. Before the formal test, each participant completed a practice phase to confirm that the stimulus–response mapping was understood. At each testing session, the formal DT was administered once. During the formal test, the Vienna Test System automatically presented visual and auditory stimuli in a randomized order and recorded the response data. The final outcome used in the analysis was the system-generated complex reaction speed value, recorded in milliseconds from the formal test; lower values indicated faster responses.

#### 2.2.2 Simple motor time

Simple motor time was assessed using the Reaction Test (RT) in the Vienna Test System. Under a simple stimulus condition, this test separates the response process into reaction time and motor time. Simple motor time represents the interval needed to convert stimulus perception into motor output and therefore reflects the efficiency of the execution stage of the response (Krzepota et al., 2015). The simple motor time testing procedure is shown in **Supplementary Figure 1B**.

After a random preparatory interval, a single visual stimulus appeared in the center of the screen. Participants released the waiting key and pressed the response key as quickly as possible after detecting the stimulus. Before the formal test, participants completed a practice phase to become familiar with the response procedure. At each testing session, the formal RT was administered once. The Vienna Test System automatically recorded both reaction time and motor time. In this study, only simple motor time was used for the main analysis. Simple motor time was defined as the movement-execution interval between releasing the waiting key and pressing the response key, and the final value was recorded in milliseconds from the system-generated formal test output. Lower values indicated faster motor execution.

#### 2.2.3 Simple lower-limb visuomotor reaction time

Simple lower-limb visuomotor reaction time was tested with the Fitlight System, based on the Reaction Time simple Lower Limb (RTsLL) protocol used in volleyball research. The test quantified the time needed to respond to a lower-limb-dominant visual stimulus, from target-light onset to foot contact and deactivation of the light. This measure captures rapid lower-limb initiation, target localization, and visuomotor response ability. Unlike traditional button-press reaction tasks, it requires stepping and foot contact with the target, and therefore better represents the action sequence common in ball sports: detecting a stimulus, moving quickly, and completing target contact. For this reason, it was used as a complementary visuomotor measure between complex reaction speed and soccer-specific reactive agility (Mancini et al., 2024). The simple lower-limb visuomotor reaction testing arrangement is shown in **Supplementary Figure 1C**.

Participants stood at the center of a semicircular setup containing eight LED lights. The starting posture required the feet to be shoulder-width apart, with the body facing the center of the testing area. The distance from the lights to the participant’s feet was standardized according to lower-limb length. When the formal test started, one target light was illuminated at random. Participants moved as fast as possible with the dominant foot and touched the corresponding light to deactivate it. Each trial lasted 45 s, and the system recorded the mean time for all valid responses. Two formal trials were completed with a 3-min rest interval, and the best average score was used for analysis.

#### 2.2.4 With-ball reactive agility time

The testing arrangement for the soccer-specific reactive agility tests is illustrated in **Supplementary Figure 2**.

With-ball reactive agility time was assessed using the Fitlight^®^ system (FITLIGHT Corp., Ontario, Canada), arranged according to soccer-specific reactive agility testing procedures. This test evaluated the ability to make directional decisions, change direction rapidly, and perform dribbling actions in response to random visual stimuli. A 5 m × 5 m square was used as the testing area, with one Fitlight target positioned at each corner. Participants began from the center while controlling the ball or preparing to dribble. The lights randomly displayed red or blue stimuli: red required deactivation with the right foot, whereas blue required deactivation with the left foot. The lights operated in proximity-sensing mode with a sensing distance of 30 cm.

At the start signal, participants began moving, after which one of the four corner lights was randomly activated. They had to identify both the position and color of the light, dribble toward the target, and deactivate it with the required foot. After one light was turned off, the next target appeared randomly, and this sequence continued until the trial was completed. Each trial included 20 random light stimuli. All lights were set to full brightness, the delay between stimuli was 0.05 s, and no timeout limit was used. The system recorded the total time needed to complete all 20 stimuli in seconds, with shorter times indicating better with-ball reactive agility.

Before formal testing, participants completed familiarization practice to ensure that they understood the light-color rules, the required response foot, and the dribbling task. Three formal trials were performed, separated by 2–3 min of rest. The best trial was retained for statistical analysis (Zwierko et al., 2024b).

#### 2.2.5 Without-ball reactive agility time

Without-ball reactive agility time was measured with the same Fitlight^®^ arrangement and stimulus protocol used for the with-ball test, but the ball-control requirement was removed. The testing area remained a 5 m × 5 m square with four Fitlight targets placed at the corners, and participants started from the center. Red or blue stimuli were presented randomly. Red required the right foot to deactivate the target light, whereas blue required the left foot. The sensing distance was maintained at 30 cm.

After the trial began, participants responded to the randomly illuminated light by making a directional judgment, initiating movement, changing direction, and reaching the target, before deactivating it with the prescribed foot. Once one target was deactivated, another light appeared randomly until 20 stimuli had been completed. The Fitlight system recorded the total completion time in seconds, and lower values indicated better without-ball reactive agility. Participants completed familiarization practice before testing, followed by three formal trials with 2–3 min of rest between trials. The best result was used for analysis (Zwierko et al., 2024b).

### 2.3 Training intervention

The stroboscopic training group and the non-stroboscopic group completed the same soccer-specific training content, with the only difference being the visual condition during training. Participants in the stroboscopic training group wore Senaptec Strobe eyewear with the stroboscopic mode activated in both lenses, whereas participants in the non-stroboscopic group wore visually similar clear non-stroboscopic eyewear that did not produce visual occlusion or stroboscopic restriction. This arrangement served as a placebo-like sham-control condition to minimize expectation effects associated with wearing specialized training equipment as much as possible. Both groups trained under the same field conditions, and training duration, content, frequency, and load were kept consistent.

Training tasks were organized around Fitlight-generated random visual stimuli and soccer-specific reactive movements. The program included three exercise categories: (a) basic visual reaction and lower-limb initiation tasks, such as starting from the center, identifying red/blue lights, touching the target with the prescribed foot, short accelerations, and rapid return to the start position; (b) off-ball reactive agility tasks, including light-triggered starts, change-of-direction movements, repeated light deactivation, and multidirectional shuttle actions in the 5 m × 5 m four-corner area; and (c) dribbling reactive agility tasks, including dribbling starts, dribbling change-of-direction actions, prescribed-foot light deactivation, continuous dribbling, and rapid return to the center under random light guidance. Each soccer-specific training session lasted approximately 25–30 min, and the exercises were conducted in the sequence of basic reaction, off-ball reactive agility, and dribbling reactive agility.

During the sessions, the stroboscopic training group used Senaptec Strobe eyewear with both lenses set in stroboscopic mode. Stroboscopic settings were configured and controlled through a mobile application. Training was arranged as repeated 2.5-min work bouts alternated with 2.5-min rest intervals. Across the 6-week period, the settings progressed as follows: 15 Hz and 50% in week 1, 13 Hz and 50% in week 2, 11 Hz and 50% in week 3, 10 Hz and 50% in week 4, 9 Hz and 60% in week 5, and 9 Hz and 70% in week 6. The intervention duration, weekly frequency, and stroboscopic settings were based mainly on earlier SVT studies and were adapted to the dribbling and off-ball reactive agility demands of soccer (Zwierko et al., 2023). This progression followed a gradual-loading principle, beginning with relatively lower visual restriction to allow adaptation and then increasing the visual challenge by reducing the strobe frequency and increasing the occlusion proportion in later weeks. The intermittent 2.5-min work and 2.5-min rest structure was used to avoid excessive visual fatigue while maintaining task difficulty.

To monitor intervention safety, participants were screened for conditions that could increase risk during stroboscopic exposure, including epilepsy, migraine, neurological disorders, severe visual impairment, and lower-limb injury. During each training session, research staff and coaches monitored participants for dizziness, visual discomfort, nausea, excessive fatigue, lower-limb pain, or other adverse symptoms. Training was paused if any discomfort occurred. Based on direct observation and participant self-report, no fatigue- or discomfort-related adverse event requiring training termination, withdrawal from the study, or medical referral was recorded during the six-week intervention. No standardized adverse-event, RPE, or visual-fatigue scale was used; therefore, safety monitoring was based on eligibility screening, direct observation, and participant self-report.

To reduce assessment bias, pre- and post-intervention tests were conducted by assessors who were blinded to group allocation. Before testing, participants were instructed not to disclose their group assignment or the type of eyewear worn during training to the assessors. Because participants could perceive whether stroboscopic occlusion was present during training, complete participant blinding was not feasible. Nevertheless, the use of non-stroboscopic clear eyewear as a sham condition, together with assessor blinding, was intended to reduce potential expectation effects and measurement bias. The weekly progression of the 6-week intervention is summarized in **Supplementary Table 1**.

### 2.4 Statistical analysis

Continuous variables were first assessed for normality using the Shapiro–Wilk test within each group, and homogeneity of variance was examined using Levene’s test. Normally distributed variables are reported as mean ± standard deviation, whereas non-normally distributed variables are reported as median [Q1, Q3]. Between-group comparisons were performed using independent-samples t-tests, Welch’s t-tests, or Mann–Whitney U tests, as appropriate. Absolute change was calculated as pre-test minus post-test, with positive values indicating improvement because lower values represented better performance for all outcomes. The primary intervention effects were evaluated using linear mixed-effects models with group, time, and the group × time interaction as fixed effects and participant ID as a random intercept. Training experience and weekly training time were included as covariates. The group × time interaction was the main parameter of interest, indicating whether the change from pre-test to post-test differed between the stroboscopic and non-stroboscopic groups. In addition, analysis of covariance was conducted for each outcome using the post-test value as the dependent variable and group as the main independent variable, adjusting for the corresponding pre-test value, training experience, and weekly training time. ANCOVA assumptions were assessed using residual normality tests, residual variance homogeneity tests, and group × baseline interaction tests. Sensitivity analyses included adjusted change-score models, HC3 robust standard errors, bootstrap confidence intervals, complete-covariate ANCOVA models, and multivariate analysis of covariance for the combined change scores. Standardized effect sizes were calculated to quantify the magnitude of between-group differences. Hedges’ g was used for change-score comparisons because of the relatively small sample size, and adjusted Cohen’s d was reported for ANCOVA-based between-group effects. Effect sizes were interpreted according to conventional thresholds, with values of approximately 0.2, 0.5, and 0.8 representing small, moderate, and large effects, respectively. A two-sided p value < 0.05 was considered statistically significant (Cohen, 1988).

## 3 Results

### 3.1 Baseline comparability

Baseline characteristics and pre-test outcome measures are presented in **Supplementary Table 2**. The two groups were comparable in demographic and training-related characteristics. No significant between-group differences were observed in age (20.261 ± 0.945 vs. 20.289 ± 0.962 years, p = 0.931), height (180.178 ± 5.901 vs. 179.333 ± 5.942 cm, p = 0.671), body weight (72.517 ± 6.074 vs. 73.483 ± 6.199 kg, p = 0.640), training experience (7.378 ± 0.817 vs. 7.428 ± 0.836 years, p = 0.857), or weekly training time (7.972 ± 0.886 vs. 8.072 ± 0.838 h/week, p = 0.730). The distributions of playing position and match status were also similar between groups, with no significant differences in playing position (p = 0.903) or match status (p = 0.738).

Pre-test outcome measures were likewise comparable between the non-stroboscopic and stroboscopic groups. There were no significant differences in simple motor time (137.439 ± 5.343 vs. 137.506± 5.439 ms, p = 0.971), complex reaction speed (733.061 ± 5.432 vs. 733.000 ± 5.546 ms, p = 0.974), lower-limb reaction time (0.599 ± 0.031 vs. 0.600 ± 0.031 s, p = 0.940), with-ball reactive agility time (62.679 ± 1.292 vs. 62.671 ± 1.319 s, p = 0.985), or without-ball reactive agility time (52.156 ± 0.835 vs. 52.156 ± 0.841 s, p = 0.998). All 95% confidence intervals for the between-group mean differences included zero, indicating that the two groups were well balanced before the intervention.

### 3.2 Post-intervention outcomes and changes from baseline

The effect sizes for change-score comparisons were moderate to large, with Hedges’ g ranging from 0.728 to 0.859, indicating consistently greater improvement in the stroboscopic group. Post-test outcomes and change scores are presented in **Supplementary Table 3**. At post-test, the stroboscopic group showed significantly lower simple motor time than the non-stroboscopic group (128.900 ± 11.138 ms vs. 136.739 ± 8.373 ms; mean difference = -7.839 ms, 95% CI: -14.513 to -1.165; t = -2.387, p = 0.023). Complex reaction speed was also significantly lower in the stroboscopic group (696.500 ± 21.971 ms vs. 713.567 ± 20.999 ms; mean difference = -17.067 ms, 95% CI: -31.624 to -2.509; U = 91.000, p = 0.026). In addition, the stroboscopic group had significantly lower with-ball reactive agility time than the non-stroboscopic group (60.822 ± 1.718 s vs. 62.429 ± 2.170 s; mean difference = -1.608 s, 95% CI: -2.934 to -0.282; t = -2.464, p = 0.019). No significant between-group differences were observed for lower-limb reaction time (0.551 ± 0.039 s vs. 0.579 ± 0.055 s; mean difference = -0.028 s, 95% CI: -0.061 to 0.004; t = -1.780, p = 0.084) or without-ball reactive agility time (51.036 ± 1.357 s vs. 51.756 ± 1.316 s; mean difference = -0.720 s, 95% CI: -1.625 to 0.185; t = -1.616, p = 0.115).

Change-score analyses showed that the stroboscopic group achieved significantly greater improvements than the non-stroboscopic group across all five outcome measures. The stroboscopic group showed greater improvement in simple motor time (8.606 ± 10.170 ms vs. 0.700 ± 8.025 ms; mean difference = 7.906 ms, 95% CI: 1.700 to 14.111; t = 2.589, p = 0.014), complex reaction speed (36.500 ± 22.847 ms vs. 19.494 ± 20.815 ms; mean difference = 17.006 ms, 95% CI: 2.201 to 31.810; t = 2.334, p = 0.026), lower-limb reaction time (0.049 ± 0.035 s vs. 0.020 ± 0.040 s; mean difference = 0.029 s, 95% CI: 0.004 to 0.054; t = 2.345, p = 0.025), with-ball reactive agility time (1.849 ± 1.799 s vs. 0.250 ± 1.841 s; mean difference = 1.599 s, 95% CI: 0.366 to 2.832; t = 2.636, p = 0.013), and without-ball reactive agility time (1.120 ± 1.017 s vs. 0.401 ± 0.913 s; mean difference = 0.719 s, 95% CI: 0.065 to 1.374; t = 2.233, p = 0.032). These results indicate that the stroboscopic group demonstrated greater pre-to-post improvements in visuomotor reaction performance and soccer-specific reactive agility.

### 3.3 Primary intervention effects based on linear mixed-effects models

Primary intervention effects were examined using linear mixed-effects models for each outcome. In each model, group, time, and the group × time interaction were entered as fixed effects, participant ID was specified as a random intercept to account for repeated measurements, and training experience and weekly training time were included as covariates. The group × time interaction was the primary term of interest because it tested whether the magnitude of pre-to-post change differed between the stroboscopic and non-stroboscopic groups. Because lower values indicated better performance for all outcomes, a negative interaction coefficient indicated a greater reduction in the stroboscopic group relative to the non-stroboscopic group.

The linear mixed-effects model results are presented in **Supplementary Table 4**. Significant group × time interactions were observed for all five outcomes. For simple motor time, the interaction effect was significant, F(1, 34.000) = 6.703, p = 0.014, with a negative interaction coefficient (β = -7.906 ms, SE = 3.053, df = 34.000, t = -2.589, 95% CI: -14.111 to -1.700). For complex reaction speed, the group × time interaction was also significant, F(1, 54.542) = 5.449, p = 0.023, β = -17.006 ms, SE = 7.285, df = 54.542, t = -2.334, 95% CI: -31.607 to -2.404. A significant interaction was also observed for lower-limb reaction time, F(1, 34.000) = 5.497, p = 0.025, β = -0.029 s, SE = 0.012, df = 34.000, t = -2.345, 95% CI: -0.054 to -0.004.

For the soccer-specific reactive agility outcomes, significant group × time interactions were found for both with-ball and without-ball conditions. For with-ball reactive agility time, the interaction effect was significant, F(1, 34.000) = 6.950, p = 0.013, β= -1.599 s, SE = 0.607, df = 34.000, t = -2.636, 95% CI: -2.832 to -0.366. For without-ball reactive agility time, the group × time interaction was also significant, F(1, 34.000) = 4.988, p = 0.032, β= -0.719 s, SE = 0.322, df = 34.000, t = -2.233, 95% CI: -1.374 to -0.065. The consistently negative interaction coefficients indicated that the stroboscopic group showed greater pre-to-post reductions than the non-stroboscopic group across all five outcomes.

### 3.4 Adjusted intervention effects based on ANCOVA

As an adjusted post-intervention comparison, analysis of covariance was performed for each outcome. In each ANCOVA model, the post-test value was used as the dependent variable, group was entered as the main independent variable, and the corresponding pre-test value, training experience, and weekly training time were included as covariates. Adjusted between-group differences were calculated as the stroboscopic group minus the non-stroboscopic group. Therefore, negative adjusted differences indicated lower adjusted post-test values in the stroboscopic group. Because lower values represented better performance for all outcomes, negative adjusted differences favoured the stroboscopic group.

The ANCOVA-adjusted results are presented in **Supplementary Table 5**. After adjustment for baseline performance and training-related covariates, the stroboscopic group showed significantly lower adjusted post-test values than the non-stroboscopic group for all five outcomes. For simple motor time, the adjusted between-group difference was -7.566 ms, SE = 3.114, df = 31, t = -2.429, F = 5.902, 95% CI: -13.918 to -1.214, p = 0.021. For complex reaction speed, the adjusted difference was -18.261 ms, SE = 7.047, df = 31, t = -2.591, F = 6.714, 95% CI: -32.635 to -3.888, p = 0.014. For lower-limb reaction time, the adjusted difference was -0.028 s, SE = 0.013, df = 31, t = -2.186, F = 4.778, 95% CI: -0.055 to -0.002, p = 0.036.

Similar adjusted between-group differences were observed for the soccer-specific reactive agility outcomes. The adjusted difference for with-ball reactive agility time was -1.624 s, SE = 0.617, df = 31, t = -2.631, F = 6.924, 95% CI: -2.882 to -0.365, p = 0.013. For without-ball reactive agility time, the adjusted difference was -0.751 s, SE = 0.332, df = 31, t = -2.263, F = 5.120, 95% CI: -1.428 to -0.074, p = 0.031. These adjusted analyses were consistent with the linear mixed-effects model results, indicating lower adjusted post-test values in the stroboscopic group after controlling for baseline performance, training experience, and weekly training time.

### 3.5 ANCOVA model diagnostics

Model assumptions for the ANCOVA analyses were evaluated before interpreting the adjusted between-group differences. Residual normality was assessed using the Shapiro–Wilk test, and homogeneity of residual variance between groups was examined using Levene’s test. The homogeneity of regression slopes assumption was assessed by adding a group × baseline outcome interaction term to each model. A non-significant interaction indicated that the relationship between baseline and post-test values did not differ significantly between groups.

The diagnostic results are shown in **Supplementary Table 6**. The residuals from all ANCOVA models showed no evidence of substantial departure from normality, with Shapiro–Wilk p values ranging from 0.211 to 0.931. Levene’s tests also indicated no evidence of unequal residual variance between groups, with p values ranging from 0.451 to 0.900. The group × baseline outcome interaction terms were not statistically significant for any outcome, including simple motor time (p = 0.762), complex reaction speed (p = 0.707), lower-limb reaction time (p = 0.082), with-ball reactive agility time (p = 0.364), and without-ball reactive agility time (p = 0.884). These results supported the use of ANCOVA for the adjusted post-intervention comparisons.

The adjusted R^2^ values ranged from 0.155 to 0.475, indicating that the explanatory ability of the ANCOVA models varied across outcomes. The models explained a relatively larger proportion of variance for without-ball reactive agility time and lower-limb reaction time, whereas the explained variance was lower for complex reaction speed. Overall, the diagnostic results suggested that the main ANCOVA assumptions were adequately satisfied, supporting the interpretation of the adjusted between-group differences.

### 3.6 Sensitivity analyses

Sensitivity analyses are presented in **Supplementary Tables 7, 8**. The direction of the intervention effects was generally consistent across adjusted change-score models, bootstrap confidence intervals, and full-covariate ANCOVA models. The multivariate analysis of the five combined change scores did not reach statistical significance. Therefore, the findings for the secondary and exploratory outcomes should be interpreted cautiously.

## 4 Discussion

This study evaluated whether stroboscopic vision training combined with soccer-specific practice could improve visuomotor reaction performance and soccer-specific reactive agility in male collegiate soccer players. Compared with the non-stroboscopic group that completed the same soccer-specific training while wearing visually similar clear non-stroboscopic eyewear, the stroboscopic training group showed larger improvements in complex reaction speed, simple motor time, simple lower-limb reaction time, and both with-ball and without-ball reactive agility time.

The stroboscopic training group showed a comparatively larger reduction in simple motor time, suggesting that the time required to complete motor output after visual-stimulus identification may have been shortened. When stroboscopic vision training (SVT) was integrated into soccer-specific training, players performed continuous actions such as receiving, first-step ball control, touch adjustment, and change-of-direction movements under restricted visual information. This training context may have encouraged players to maintain motor readiness under incomplete visual information and to organize subsequent actions based on key cues. When visual input was restored, this adaptation may have been behaviorally expressed as faster motor output. Previous studies have shown that SVT can have positive effects on sport tasks involving rapid visual-cue identification, visual tracking, and motor execution, and systematic reviews have also reported beneficial effects on hand–eye coordination, reaction time, agility, and visuomotor performance (Wilkins and Gray, 2015; Wang et al., 2025). Therefore, the reduction in simple motor time may not only be related to motor execution itself, but may also reflect improved efficiency in the linkage between visual-cue extraction, motor preparation, and motor output. This interpretation can be understood with reference to physiological evidence on early visual processing: P100 is commonly regarded as an early index of sensory processing in the visual cortical system following visual-stimulus presentation, and changes in its latency are related to the timing of early visual information transmission (Di Russo et al., 2002). Accordingly, the decrease in simple motor time in the present study may be cautiously interpreted as a behavioral manifestation of improved motor-output efficiency after stimulus identification.

The present results showed that the stroboscopic training group exhibited a comparatively larger reduction in simple lower-limb visuomotor reaction time, indicating that the time required to complete lower-limb initiation, target-directed movement, and foot contact after the appearance of a random visual cue may have been shortened. Unlike simple motor time, this test involves a sequence of actions, including body orientation, center-of-mass transfer, lower-limb initiation, and foot contact, making it more closely related to the demands of acceleration, change of direction, and first-step ball control in soccer (Pojskic et al., 2018; Jothi et al., 2025). When stroboscopic training was embedded into soccer-specific practice, players were required to complete receiving, touch adjustment, change-of-direction, and first-step ball-control actions under restricted visual input. This may have strengthened the functional coupling between visual stimuli, lower-limb initiation, directional adjustment, and foot contact. Consistent with this interpretation, previous volleyball research reported that 6 weeks of SVT improved visuomotor reaction speed during goal-directed actions in young athletes, providing indirect support for the possibility that stroboscopic training may enhance target-directed lower-limb responses (Zwierko et al., 2024a). In this process, feedforward motor control, postural pre-adjustment, and the integration of visual information with proprioceptive feedback may help athletes organize the lower-limb action chain more rapidly after the appearance of random visual cues (Appelbaum and Erickson, 2018; Wilkins and Appelbaum, 2020; Symeonidou and Ferris, 2022).

The present results showed that the stroboscopic training group exhibited a comparatively greater improvement in complex reaction speed, suggesting that the efficiency of stimulus identification, attentional allocation, response selection, and motor output in a rapidly changing multi-stimulus context may have been enhanced. Unlike the single-stimulus response involved in simple motor time, complex reaction speed includes multiple processing stages, such as multi-stimulus identification, allocation of attentional resources, response selection, and motor execution (Teteris et al., 2025). Compared with reaction practice under normal visual conditions, SVT increases information uncertainty in multi-cue tasks and requires athletes to complete effective cue extraction and stimulus–response mapping more rapidly when visual input is intermittent. This training demand may help improve visuomotor processing efficiency in complex reaction tasks. This change may further reflect improved processing efficiency during complex stimulus–response mapping. In previous neurophysiological research, the N2 component has often been used to reflect pre-motor processes such as stimulus evaluation, conflict monitoring, and response selection, and therefore provides a possible theoretical explanation for the improvement in complex reaction speed (Folstein and Van Petten, 2008). Relevant reviews and meta-analyses have indicated that SVT may have potential positive effects on reaction time and visuomotor performance, but its effects are influenced by training duration, task characteristics, assessment methods, and the transfer distance between training and testing (Wilkins and Appelbaum, 2020; Luo et al., 2025; Wang et al., 2025). Therefore, the improvement in complex reaction speed observed in the present study is better understood as a result of the close match between stroboscopic visual restriction and soccer-specific multi-cue reaction tasks, rather than as evidence that SVT generally enhances all visual-cognitive abilities. Soccer vision-training research also suggests that training effects may be task-selective and may not necessarily produce concurrent changes across all perceptual-cognitive variables (Fortes et al., 2025).

In the soccer-specific reactive agility test, the stroboscopic training group showed a small but comparatively larger reduction in with-ball reactive agility time. Reactive agility depends not only on change-of-direction speed, but also on the ability to perceive external cues, make decisions, and execute actions (Sheppard and Young, 2006; Young and Willey, 2010). In soccer, with-ball contexts further require players to make directional judgments and adjust their actions while maintaining ball control, thereby better reflecting the perception–action coupling characteristics of match-like situations (Pojskic et al., 2018). Participants in the stroboscopic group repeatedly completed the task sequence of “random light stimulus–color identification–prescribed-foot light deactivation–continuous change-of-direction movements–with-ball execution.” This training structure was highly consistent with the with-ball reactive agility test in terms of visual-cue identification, action organization, and soccer-specific context, thereby providing a basis for the transfer of training effects to the with-ball reactive agility task. More importantly, SVT did not simply involve repeating the same movements; rather, by intermittently occluding visual input, it increased the demand for players to maintain ball control, anticipate direction, and adjust actions under incomplete information. Therefore, players may have become faster at using key visual cues during dribbling while simultaneously organizing step adjustments, touch rhythm, and change-of-direction paths, thereby strengthening the functional coupling among visual cues, ball-control actions, and change-of-direction execution. Consistent with this interpretation, previous soccer research has shown that stroboscopic eyewear can improve with-ball reactive agility performance, whereas its effect on without-ball reactive agility is relatively limited, suggesting that the training benefits of SVT may depend more strongly on the integration of visual information extraction, technical control, and motor execution in ball-control tasks (Zwierko et al., 2024b).

Although without-ball reactive agility time also decreased in the stroboscopic training group, the size of the improvement was relatively modest. This task requires external-stimulus identification, response selection, and directional change, but it does not involve ball contact, ball control, or dribbling adjustment. It may therefore represent general acceleration, reactive movement ability, and change-of-direction execution to a greater extent (Young and Willey, 2010). Previous research has emphasized that reactive agility and pre-planned change-of-direction speed are distinct abilities. In soccer, adding ball-control demands increases the need for sport-specific technique and perception–action coupling (Spasic et al., 2015). Thus, the stronger advantage observed for the with-ball task was more likely related to the similarity between the training and testing constraints than to a general improvement in speed or agility alone.

### 4.1 Embedded SVT and practical implications for team sports

The present study suggests that SVT may serve as an auxiliary visual constraint within regular soccer-specific training, rather than as an isolated visual training method detached from sport-specific contexts (Dingirdan Gultekinler et al., 2025). In the present study, both groups completed the same soccer-specific training content, training frequency, and training load, with the only difference being that the stroboscopic group received intermittent visual restriction during training. Therefore, the comparatively greater improvements observed in visuomotor reaction and soccer-specific reactive-agility outcomes in the stroboscopic group may be interpreted as additional training benefits of SVT beyond regular soccer training. This finding has certain implications for invasion team sports played in shared spaces. In these sports, athletes must rapidly identify external cues and complete action selection, body adjustment, and attack–defense transitions while teammates, opponents, the ball, and spatial positions are continuously changing (Paul et al., 2016; Gudmundsson and Horton, 2018). Thus, the applied value of SVT may lie primarily in improving athletes’ ability to extract visual cues, adjust actions, and respond to dynamic situations under incomplete visual information, rather than in simply enhancing general visual function. In the present study, improvements in reaction-time-related measures suggest that basic visuomotor processing efficiency may have improved, whereas reductions in with-ball and without-ball reactive agility times suggest that this improvement may have been further expressed as faster situational responses in soccer-specific contexts. Similarly, team ball-sport research has shown that embedding SVT into sport-specific training can improve visuomotor reaction speed and reactive agility performance during goal-directed actions (Zwierko et al., 2024a). In addition, reviews of sports vision research indicate that the training context plays an important role in the transfer of visual training to sport-specific skills, and that visual training detached from sport-specific contexts may be more likely to improve general cognitive performance than to sufficiently enhance sport-specific technical performance (Buscemi et al., 2024). Therefore, coaches may use SVT as an auxiliary strategy within regular training by embedding it into sport-specific drills that require rapid identification of external cues, technical-action adjustment, and situational responses, with the aim of improving athletes’ movement organization, response capability, and rapid visuomotor transformation ability in dynamic environments (Nimmerichter et al., 2016; Zwierko et al., 2024a). However, SVT should be implemented in a short-duration, progressive, and task-embedded manner, while ensuring technical movement quality and training safety.

### 4.2 Limitations and future research

The present findings suggest that stroboscopic vision training may help improve visuomotor reaction ability and soccer-specific reactive agility performance in male collegiate soccer players, but several limitations should be noted. First, training effects were mainly evaluated using laboratory-based visuomotor reaction tests and with-ball/without-ball reactive agility tests, while match-performance indicators were not included. Therefore, the present results mainly indicate that stroboscopic vision training (SVT) may improve visuomotor reaction ability and sport-specific reactive agility performance under controlled testing conditions; however, whether these improvements can be effectively transferred to skill performance in actual match situations remains unclear. Second, the present study did not collect neurophysiological, eye-tracking, or biomechanical measures. Thus, although the behavioral results showed comparatively greater improvements in visuomotor reaction and soccer-specific reactive agility outcomes in the stroboscopic training group, this study cannot directly determine whether these changes were related to alterations in neurophysiological processing, visual search behavior, neuromuscular control, or kinematic mechanisms. Third, the sample included only male collegiate soccer players; therefore, whether the findings can be generalized to athletes of different sexes, ages, and competitive levels requires further verification. Fourth, although the non-stroboscopic group wore visually similar clear eyewear to reduce expectation effects, complete participant blinding was not feasible because participants could perceive whether stroboscopic occlusion was present during training. Therefore, potential expectation effects and motivation-related influences cannot be completely excluded. Fifth, safety monitoring mainly relied on eligibility screening, direct observation, and participant self-report, rather than standardized adverse-event recording or visual-fatigue scales; this limitation should be addressed in future research.

Future research should recruit more diverse samples, include retention assessments, and use stricter control designs. Studies could also combine match-like soccer outcomes with eye-tracking, electroencephalography, or motion-capture methods to clarify how SVT influences soccer reactive performance and sport-specific ability.

## 5 Conclusions

The present findings show that, compared with the same soccer-specific training performed with visually similar clear non-stroboscopic eyewear, 6 weeks of stroboscopic vision training combined with soccer-specific practice was associated with larger improvements in simple motor time, lower-limb reaction time, complex reaction speed, and both with-ball and without-ball reactive agility performance in male collegiate soccer players. These results suggest that SVT may provide additional short-term benefits when integrated into soccer-specific reactive training. This approach may be used as a supplementary method for developing soccer-specific reactive ability; nevertheless, whether the effects are retained over time and transferred to actual match performance requires further verification.

## Data Availability

The original contributions presented in the study are included in the article/**Supplementary Material**, further inquiries can be directed to the corresponding author/s.
