# Supplementary material for: Effects of stroboscopic vision training on visuomotor reaction and soccer-specific reactive agility in male collegiate soccer players: a randomized controlled intervention study
Source: Front Physiol. 2026 Jul 20;17:1897512. doi: 10.3389/fphys.2026.1897512 (PMC13429501; doi:10.3389/fphys.2026.1897512)
Supplement: Supplementary file 1 [file SupplementaryFile1.docx]

Supplementary Material

# Supplementary Figures and Tables

## Supplementary Figures

**
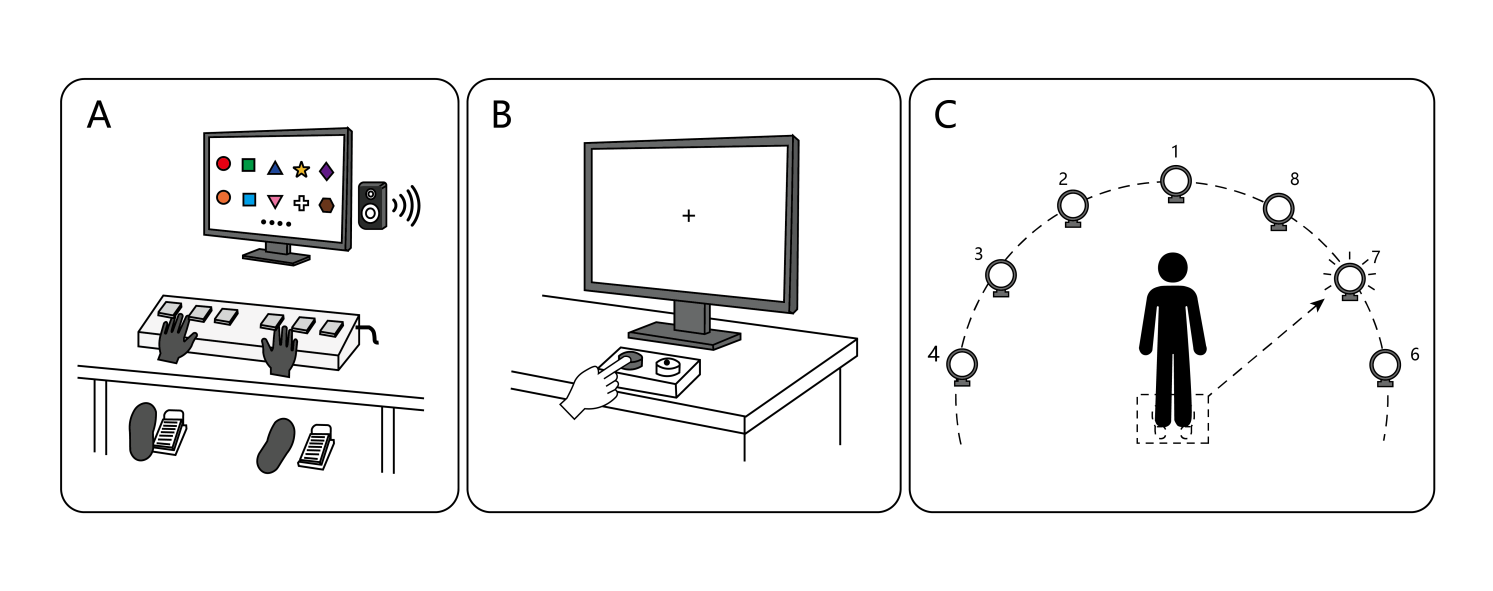
**

**Figure 1.** (A) Complex reaction speed test using the Determination Test in the Vienna Test System. Participants responded to rapidly changing visual and auditory stimuli using the corresponding hand keys or foot pedals. Schematic illustration of the laboratory-based visuomotor reaction tests. (B) Simple motor time test using the Reaction Test in the Vienna Test System. Participants released the waiting key and pressed the response key as quickly as possible after detecting a single visual stimulus.(C) Simple lower-limb visuomotor reaction time test using the Fitlight System. Participants stood in the center of a semicircular light arrangement and deactivated the randomly illuminated target with the dominant foot. The figure is a schematic illustration and is not drawn to scale.

| A.With-ball reactive agility time  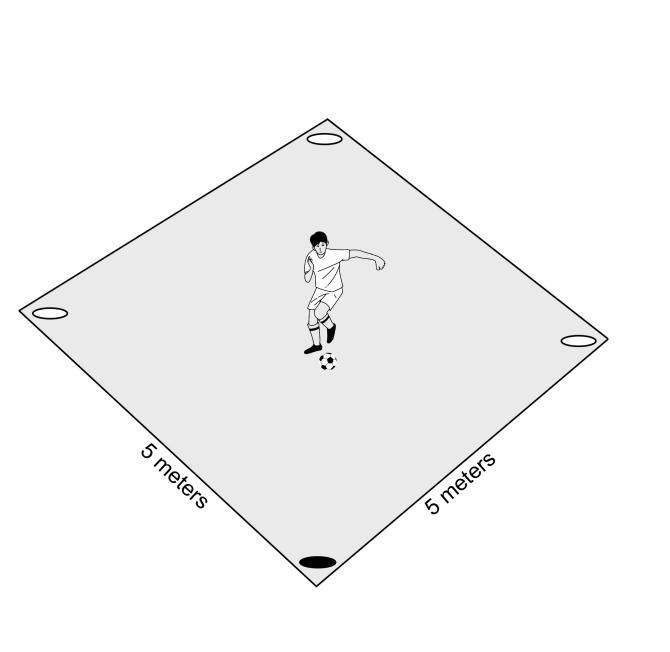 | B.Without-ball reactive agility time  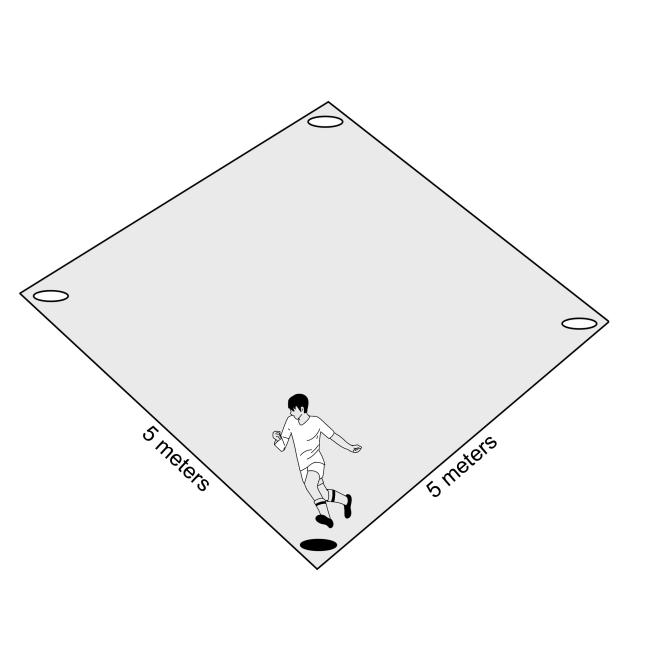 |
| --- | --- |

**Figure 2.** Testing arrangement for soccer-specific reactive agility. (A) With-ball reactive agility time test. (B) Without-ball reactive agility time test. Four Fitlight targets were positioned at the corners of a 5 m × 5 m square, and participants began from the center. In both conditions, participants responded to randomly presented red or blue light stimuli and deactivated the target using the prescribed foot. The without-ball test followed the same procedure as the with-ball test, except that ball control was not required.

**
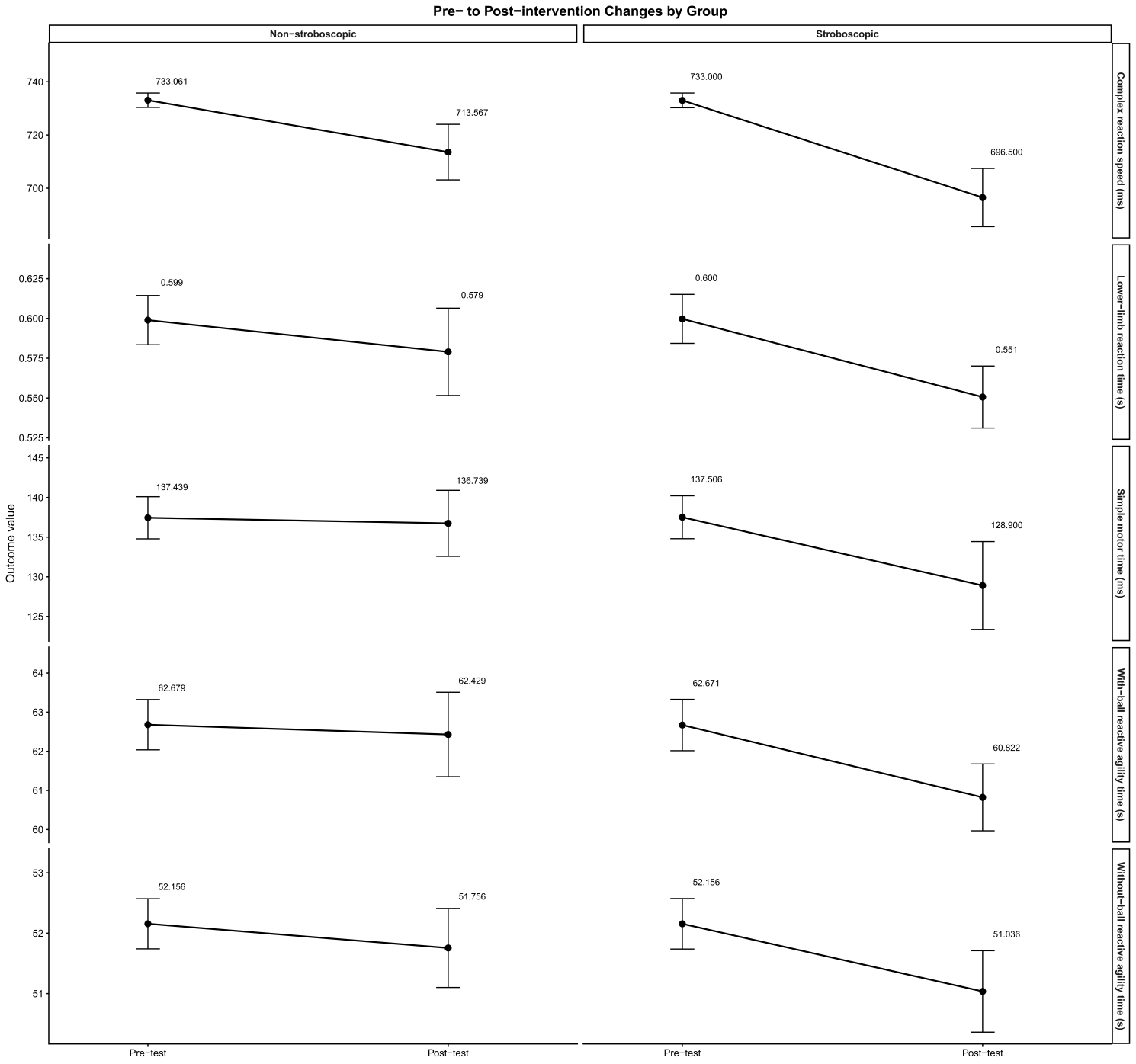
**

**Figure 3.** Pre-to-post intervention changes in outcome measures by group. Points represent group means, and error bars represent 95% confidence intervals. Lower-limb reaction time refers to simple lower-limb visuomotor reaction time.

**
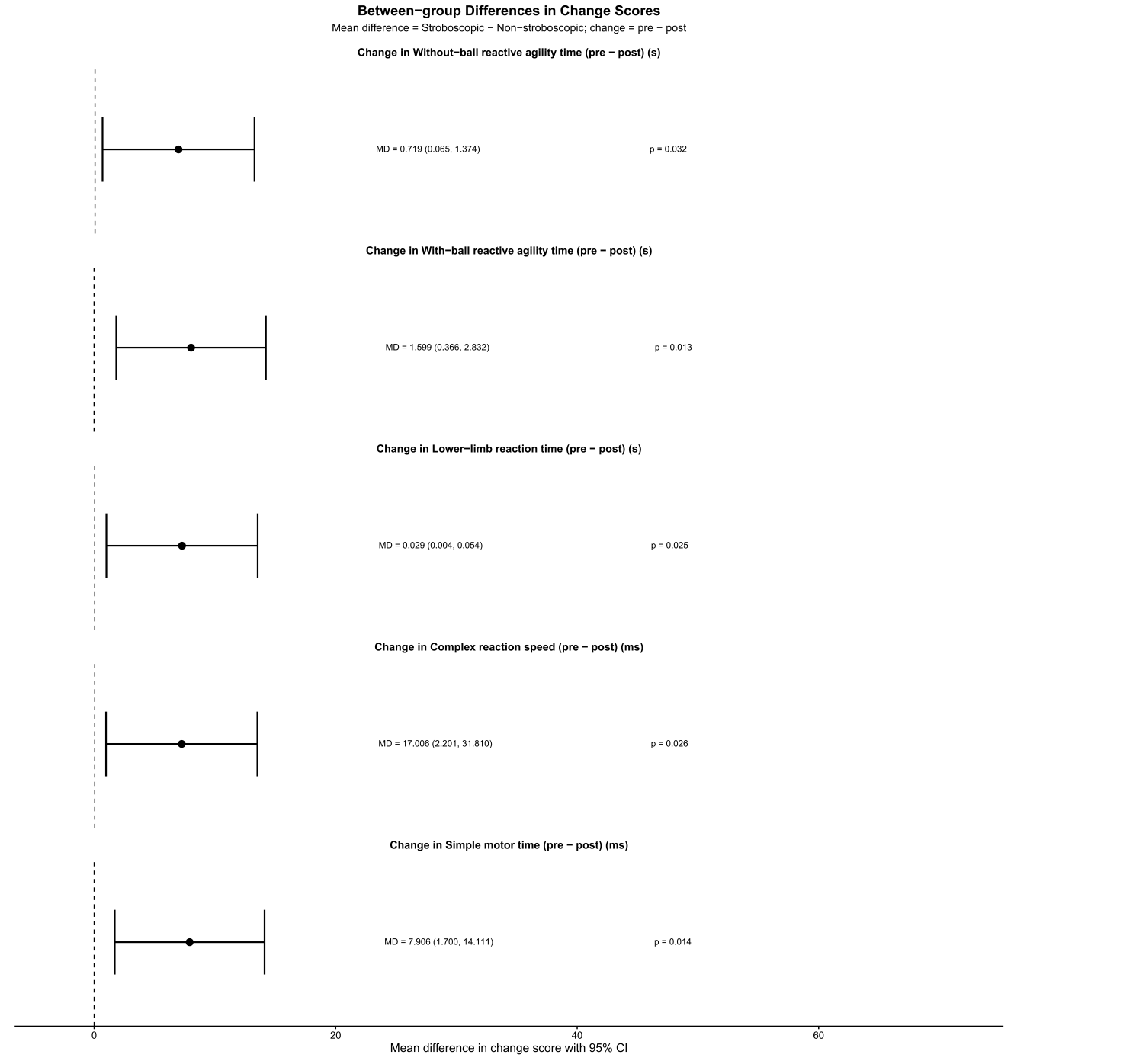
**

**Figure 4.** Between-group differences in pre-to-post change scores. Change scores were calculated as pre-test minus post-test. Points represent mean differences, and error bars represent 95% confidence intervals. Positive values indicate greater improvement in the stroboscopic group.

**
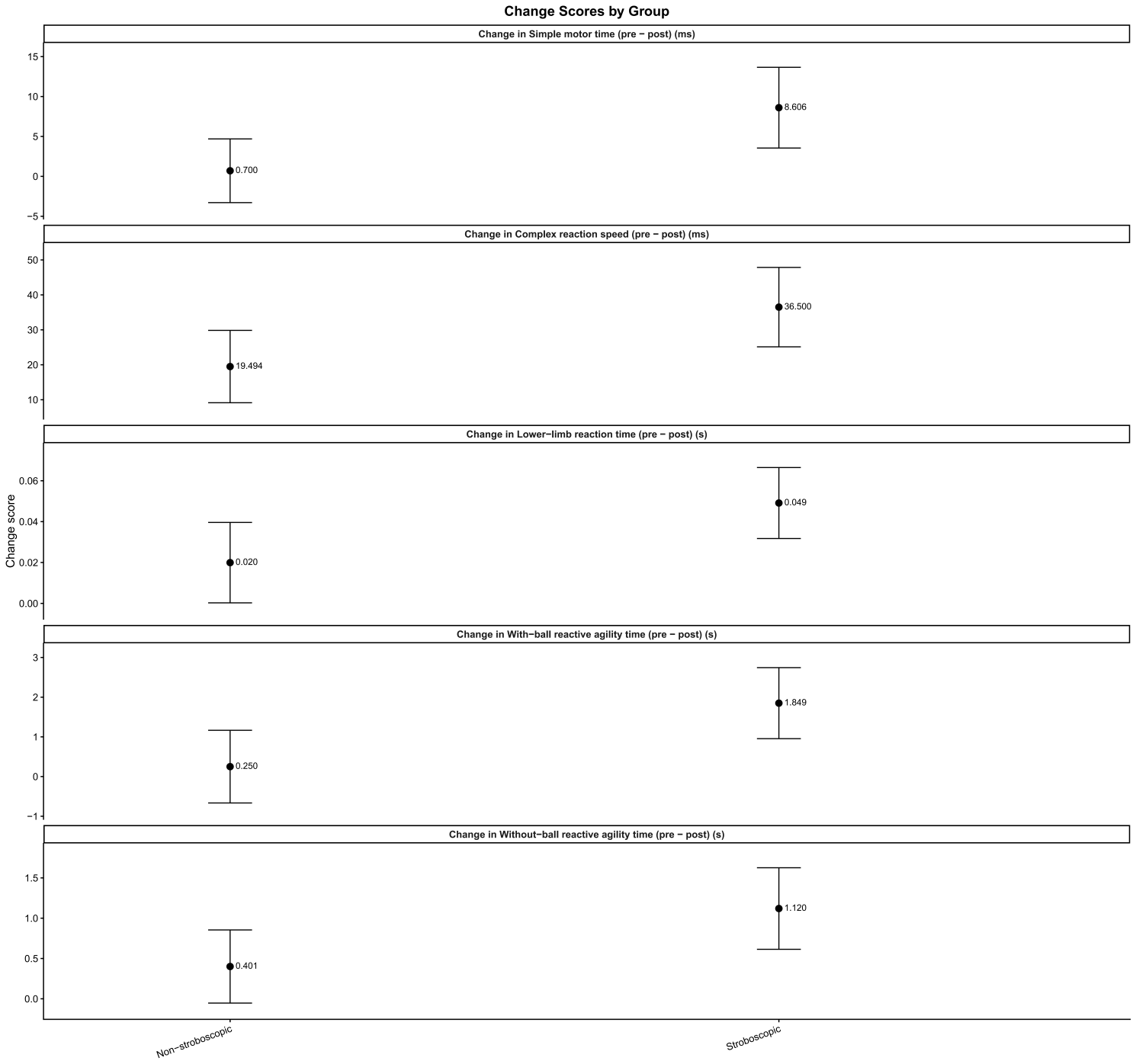
**

**Figure 5.** Change scores from pre-test to post-test by group. Change scores were calculated as pre-test minus post-test; positive values indicate improvement. Points represent group means, and error bars represent 95% confidence intervals.

## Supplementary Tables

**Table 1.** Weekly progression of the 6-week soccer-specific stroboscopic vision training intervention.

| **Week** | **Training schedule** | **Task organization and progression emphasis** | **Work/rest structure** | **Stroboscopic group setting** | **Non-stroboscopic group setting** |
| --- | --- | --- | --- | --- | --- |
| Week 1 | 3 sessions/week; approximately 25–30 min/session | Familiarization with the training sequence and stroboscopic visual environment; emphasis on basic visual reaction, lower-limb initiation, and basic off-ball reactive movements while maintaining movement quality and task understanding | Repeated 2.5-min work bouts alternated with 2.5-min rest intervals | 15 Hz; 50% occlusion proportion | Visually similar clear non-stroboscopic eyewear; no visual occlusion or stroboscopic restriction |
| Week 2 | 3 sessions/week; approximately 25–30 min/session | Continued basic visual reaction and lower-limb initiation tasks, with increased continuity of short accelerations, prescribed-foot light deactivation, and rapid return movements | Repeated 2.5-min work bouts alternated with 2.5-min rest intervals | 13 Hz; 50% occlusion proportion | Visually similar clear non-stroboscopic eyewear; no visual occlusion or stroboscopic restriction |
| Week 3 | 3 sessions/week; approximately 25–30 min/session | Continued implementation of the three task categories, with greater emphasis on direction judgment and change-of-direction execution under Fitlight-generated random visual stimuli | Repeated 2.5-min work bouts alternated with 2.5-min rest intervals | 11 Hz; 50% occlusion proportion | Visually similar clear non-stroboscopic eyewear; no visual occlusion or stroboscopic restriction |
| Week 4 | 3 sessions/week; approximately 25–30 min/session | Greater emphasis on off-ball reactive agility, including light-triggered starts, repeated light deactivation, and multidirectional shuttle actions, while maintaining controlled dribbling task execution | Repeated 2.5-min work bouts alternated with 2.5-min rest intervals | 10 Hz; 50% occlusion proportion | Visually similar clear non-stroboscopic eyewear; no visual occlusion or stroboscopic restriction |
| Week 5 | 3 sessions/week; approximately 25–30 min/session | Increased visual uncertainty and continuous movement organization during off-ball and dribbling reactive agility tasks | Repeated 2.5-min work bouts alternated with 2.5-min rest intervals | 9 Hz; 60% occlusion proportion | Visually similar clear non-stroboscopic eyewear; no visual occlusion or stroboscopic restriction |
| Week 6 | 3 sessions/week; approximately 25–30 min/session | Highest planned visual restriction level; completion of basic reaction, off-ball reactive agility, and dribbling reactive agility tasks under random light guidance, with emphasis on movement quality and safe task execution | Repeated 2.5-min work bouts alternated with 2.5-min rest intervals | 9 Hz; 70% occlusion proportion | Visually similar clear non-stroboscopic eyewear; no visual occlusion or stroboscopic restriction |

**Table 1 Note.** Hz = stroboscopic frequency. Occlusion proportion refers to the visual restriction setting configured through the Senaptec Strobe application. Both groups completed the same training content, frequency, duration, workload, and exercise sequence; only the visual condition differed.

**Table 2.** Baseline characteristics of the participants.

| **Variable** | **Non-stroboscopic group (n = 18)** | **Stroboscopic group (n = 18)** | **Mean difference (95% CI)** | **Test** | **Statistic** | **p value** |
| --- | --- | --- | --- | --- | --- | --- |
| **Baseline characteristics** | | | | | | |
| Age (years) | 20.261 ± 0.945 | 20.289 ± 0.962 | 0.028 (-0.618, 0.674) | Independent-samples t-test | t = 0.087 | 0.931 |
| Height (cm) | 180.178 ± 5.901 | 179.333 ± 5.942 | -0.844 (-4.856, 3.167) | Independent-samples t-test | t = -0.428 | 0.671 |
| Weight (kg) | 72.517 ± 6.074 | 73.483 ± 6.199 | 0.967 (-3.190, 5.124) | Independent-samples t-test | t = 0.473 | 0.640 |
| Training experience (years) | 7.378 ± 0.817 | 7.428 ± 0.836 | 0.050 (-0.510, 0.610) | Independent-samples t-test | t = 0.181 | 0.857 |
| Weekly training time (h/week) | 7.972 ± 0.886 | 8.072 ± 0.838 | 0.100 (-0.484, 0.684) | Independent-samples t-test | t = 0.348 | 0.730 |
| Playing position, n (%) |  |  | — | Fisher–Freeman–Halton exact test | — | 0.903 |
| — Defender | 7 (38.9) | 9 (50.0) | — |  |  |  |
| — Midfielder | 8 (44.4) | 6 (33.3) |  |  |  |  |
| — Forward | 3 (16.7) | 3 (16.7) |  |  |  |  |
| Match status, n (%) |  |  |  | Fisher’s exact test | — | 0.738 |
| — Starter | 9 (50.0) | 7 (38.9) |  |  |  |  |
| — Substitute | 9 (50.0) | 11 (61.1) |  |  |  |  |
| **Baseline outcome measures** | | | | | | |
| Simple motor time at pre-test (ms) | 137.439 ± 5.343 | 137.506 ± 5.439 | 0.067 (-3.585, 3.719) | Independent-samples t-test | t = 0.037 | 0.971 |
| Complex reaction speed at pre-test (ms) | 733.061 ± 5.432 | 733.000 ± 5.546 | -0.061 (-3.780, 3.658) | Independent-samples t-test | t = -0.033 | 0.974 |
| Lower-limb reaction time at pre-test (s) | 0.599 ± 0.031 | 0.600 ± 0.031 | 0.001 (-0.020, 0.022) | Independent-samples t-test | t = 0.075 | 0.940 |
| With-ball reactive agility time at pre-test (s) | 62.679 ± 1.292 | 62.671 ± 1.319 | -0.008 (-0.893, 0.876) | Independent-samples t-test | t = -0.019 | 0.985 |
| Without-ball reactive agility time at pre-test (s) | 52.156 ± 0.835 | 52.156 ± 0.841 | -0.001 (-0.568, 0.567) | Independent-samples t-test | t = -0.002 | 0.998 |

**Table 2 Note.** Values are presented as mean ± standard deviation or n (%). Mean differences were calculated as the stroboscopic group minus the non-stroboscopic group. CI, confidence interval. Lower-limb reaction time refers to simple lower-limb visuomotor reaction time.

**Table 3.** Post-intervention outcomes and changes from baseline between groups

| **Outcome** | **Post-test Non-stroboscopic group** | **Post-test Stroboscopic group** | **Post-test difference 95% CI** | **Post-test test statistic** | **Post-test p** | **Change Non-stroboscopic group** | **Change Stroboscopic group** | **Change difference 95% CI** | **Change test statistic** | **Change p** |
| --- | --- | --- | --- | --- | --- | --- | --- | --- | --- | --- |
| Simple motor time ms | 136.739 ± 8.373 | 128.900 ± 11.138 | -7.839 -14.513 to -1.165 | t = -2.387, df = 34 | 0.023 | 0.700 ± 8.025 | 8.606 ± 10.170 | 7.906 1.700 to 14.111 | t = 2.589, df = 34 | 0.014 |
| Complex reaction speed ms | 713.567 ± 20.999 | 696.500 ± 21.971 | -17.067 -31.624 to -2.509 | U = 91.000 | 0.026 | 19.494 ± 20.815 | 36.500 ± 22.847 | 17.006 2.201 to 31.810 | t = 2.334, df = 34 | 0.026 |
| Lower-limb reaction time s | 0.579 ± 0.055 | 0.551 ± 0.039 | -0.028 -0.061 to 0.004 | t = -1.780, df = 34 | 0.084 | 0.020 ± 0.040 | 0.049 ± 0.035 | 0.029 0.004 to 0.054 | t = 2.345, df = 34 | 0.025 |
| With-ball reactive agility time s | 62.429 ± 2.170 | 60.822 ± 1.718 | -1.608 -2.934 to -0.282 | t = -2.464, df = 34 | 0.019 | 0.250 ± 1.841 | 1.849 ± 1.799 | 1.599 0.366 to 2.832 | t = 2.636, df = 34 | 0.013 |
| Without-ball reactive agility time s | 51.756 ± 1.316 | 51.036 ± 1.357 | -0.720 -1.625 to 0.185 | t = -1.616, df = 34 | 0.115 | 0.401 ± 0.913 | 1.120 ± 1.017 | 0.719 0.065 to 1.374 | t = 2.233, df = 34 | 0.032 |

**Table 3 Note.** Values are presented as mean ± standard deviation. Change scores were calculated as pre-test minus post-test; therefore, positive values indicate improvement. Differences were calculated as the stroboscopic group minus the non-stroboscopic group. CI, confidence interval.

**Table 4.** Linear mixed-effects model results for the group × time interaction.

| **Outcome** | **β for group × time interaction (95% CI)** | **SE** | **df** | **t value** | **p value** | **Holm-adjusted p** | **FDR q** |
| --- | --- | --- | --- | --- | --- | --- | --- |
| Simple motor time (ms) | -7.906 (-14.111, -1.700) | 3.053 | 34.000 | -2.589 | 0.014 | 0.063 | 0.031 |
| Complex reaction speed (ms) | -17.006 (-31.607, -2.404) | 7.285 | 54.542 | -2.334 | 0.023 | 0.070 | 0.031 |
| Lower-limb reaction time (s) | -0.029 (-0.054, -0.004) | 0.012 | 34.000 | -2.345 | 0.025 | 0.070 | 0.031 |
| With-ball reactive agility time (s) | -1.599 (-2.832, -0.366) | 0.607 | 34.000 | -2.636 | 0.013 | 0.063 | 0.031 |
| Without-ball reactive agility time (s) | -0.719 (-1.374, -0.065) | 0.322 | 34.000 | -2.233 | 0.032 | 0.070 | 0.032 |

**Table 4 Note.** β represents the group × time interaction effect from the linear mixed-effects model. Negative β values indicate greater reductions in the stroboscopic group. CI, confidence interval; SE, standard error; FDR, false discovery rate.

**Table 5.** ANCOVA-adjusted between-group differences in post-intervention outcomes.

| **Outcome** | **Adjusted difference 95% CI** | **SE** | **df** | **t value** | **F value** | **p value** | **Holm-adjusted p** | **FDR q** |
| --- | --- | --- | --- | --- | --- | --- | --- | --- |
| Simple motor time (ms) | -7.566 (-13.918, -1.214) | 3.114 | 31 | -2.429 | 5.902 | 0.021 | 0.066 | 0.035 |
| Complex reaction speed (ms) | -18.261 (-32.635, -3.888) | 7.047 | 31 | -2.591 | 6.714 | 0.014 | 0.066 | 0.035 |
| Lower-limb reaction time (s) | -0.028 (-0.055, -0.002) | 0.013 | 31 | -2.186 | 4.778 | 0.036 | 0.066 | 0.036 |
| With-ball reactive agility time (s) | -1.624 (-2.882, -0.365) | 0.617 | 31 | -2.631 | 6.924 | 0.013 | 0.066 | 0.035 |
| Without-ball reactive agility time (s) | -0.751 (-1.428, -0.074) | 0.332 | 31 | -2.263 | 5.120 | 0.031 | 0.066 | 0.036 |

**Table 5 Note.** ANCOVA models were adjusted for the corresponding pre-test value, training experience, and weekly training time. Adjusted differences were calculated as the stroboscopic group minus the non-stroboscopic group. CI, confidence interval; SE, standard error; FDR, false discovery rate.

**Table 6.** ANCOVA diagnostic results and homogeneity of regression slopes.

| **Outcome** | **Residual Shapiro–Wilk p** | **Residual Levene p** | **Adjusted R²** | **Homogeneity test** | **F value** | **p value** |
| --- | --- | --- | --- | --- | --- | --- |
| Simple motor time (ms) | 0.931 | 0.451 | 0.215 | Group × pre-test value | 0.093 | 0.762 |
| Complex reaction speed (ms) | 0.470 | 0.857 | 0.155 | Group × pre-test value | 0.144 | 0.707 |
| Lower-limb reaction time (s) | 0.211 | 0.672 | 0.381 | Group × pre-test value | 3.238 | 0.082 |
| With-ball reactive agility time (s) | 0.345 | 0.900 | 0.227 | Group × pre-test value | 0.851 | 0.364 |
| Without-ball reactive agility time (s) | 0.577 | 0.670 | 0.475 | Group × pre-test value | 0.022 | 0.884 |

**Table 6 Note.** Residual normality, residual variance homogeneity, and homogeneity of regression slopes were assessed using the Shapiro–Wilk test, Levene’s test, and group × pre-test value interaction, respectively.

**Table 7.** Sensitivity analyses of intervention effect

| Outcome | Adjusted change-score model β (95% CI), p | HC3 robust β, p | Bootstrap mean effect, BCa 95% CI | Full-covariate ANCOVA β (95% CI), p |
| --- | --- | --- | --- | --- |
| Simple motor time (ms) | 7.566 (1.214 to 13.918), p = 0.021 | 7.566, p = 0.031 | -7.330 (-13.302 to -1.109) | -7.255 (-15.118 to 0.609), p = 0.069 |
| Complex reaction speed (ms) | 18.261 (3.888 to 32.635), p = 0.014 | 18.261, p = 0.017 | -17.739 (-31.529 to -4.874) | -21.648 (-39.952 to -3.344), p = 0.022 |
| Lower-limb reaction time (s) | 0.028 (0.002 to 0.055), p = 0.036 | 0.028, p = 0.055 | -0.027 (-0.056 to -0.003) | -0.032 (-0.066 to 0.002), p = 0.061 |
| With-ball reactive agility time (s) | 1.624 (0.365 to 2.882), p = 0.013 | 1.624, p = 0.019 | -1.605 (-2.844 to -0.397) | -2.359 (-3.849 to -0.869), p = 0.003 |
| Without-ball reactive agility time (s) | 0.751 (0.074 to 1.428), p = 0.031 | 0.751, p = 0.043 | -0.729 (-1.390 to -0.100) | -0.967 (-1.764 to -0.171), p = 0.019 |

**Table 7 Note.** Sensitivity analyses included adjusted change-score models, HC3 robust standard errors, bootstrap confidence intervals, and full-covariate ANCOVA models. HC3, heteroscedasticity-consistent type 3 standard error; BCa, bias-corrected and accelerated bootstrap confidence interval; CI, confidence interval.

**Table 8.** Multivariate analysis of covariance for combined change scores.

| Term | Test | Statistic | Approx. F | Num df | Den df | p value |
| --- | --- | --- | --- | --- | --- | --- |
| Group | Pillai’s trace | 0.280 | 2.175 | 5 | 28 | 0.086 |
| Training experience | Pillai’s trace | 0.096 | 0.597 | 5 | 28 | 0.703 |
| Weekly training time | Pillai’s trace | 0.198 | 1.382 | 5 | 28 | 0.261 |
| Group | Wilks’ lambda | 0.720 | 2.175 | 5 | 28 | 0.086 |
| Training experience | Wilks’ lambda | 0.904 | 0.597 | 5 | 28 | 0.703 |
| Weekly training time | Wilks’ lambda | 0.802 | 1.382 | 5 | 28 | 0.261 |

**Table 8 Note.** Multivariate analysis of covariance was performed for the combined change scores, with group as the main factor and training experience and weekly training time as covariates. Approx. F, approximate F statistic; Num df, numerator degrees of freedom; Den df, denominator degrees of freedom.
